# Rapid enzyme regeneration results in the striking catalytic longevity of an engineered, single species, biocatalytic biofilm

**DOI:** 10.1186/s12934-016-0579-3

**Published:** 2016-10-21

**Authors:** Xiaoxue Tong, Tania Triscari Barberi, Catherine H. Botting, Sunil V. Sharma, Mark J. H. Simmons, Tim W. Overton, Rebecca J. M. Goss

**Affiliations:** 1 School of Chemistry, University of St. Andrews, St. Andrews, KY16 9ST UK; 2 Biomedical Sciences Research Complex, University of St. Andrews, St. Andrews, KY16 9ST UK; 3 School of Chemical Engineering, University of Birmingham, Edgbaston, Birmingham, B152TT UK

**Keywords:** Engineered *E. coli* biofilm, Biotransformation, Biocatalysis, Tryptophan synthase, Halotryptophan, SILAC, Proteomic analysis

## Abstract

**Background:**

Engineering of single-species biofilms for enzymatic generation of fine chemicals is attractive. We have recently demonstrated the utility of an engineered *Escherichia coli* biofilm as a platform for synthesis of 5-halotryptophan. *E. coli* PHL644, expressing a recombinant tryptophan synthase, was employed to generate a biofilm. Its rapid deposition, and instigation of biofilm formation, was enforced by employing a spin-down method. The biofilm presents a large three-dimensional surface area, excellent for biocatalysis. The catalytic longevity of the engineered biofilm is striking, and we had postulated that this was likely to largely result from protection conferred to recombinant enzymes by biofilm’s extracellular matrix. SILAC (stable isotopic labelled amino acids in cell cultures), and in particular dynamic SILAC, in which pulses of different isotopically labelled amino acids are administered to cells over a time course, has been used to follow the fate of proteins. To explore within our spin coated biofilm, whether the recombinant enzyme’s longevity might be in part due to its regeneration, we introduced pulses of isotopically labelled lysine and phenylalanine into medium overlaying the biofilm and followed their incorporation over the course of biofilm development.

**Results:**

Through SILAC analysis, we reveal that constant and complete regeneration of recombinant enzymes occurs within spin coated biofilms. The striking catalytic longevity within the biofilm results from more than just simple protection of active enzyme by the biofilm and its associated extracellular matrix. The replenishment of recombinant enzyme is likely to contribute significantly to the catalytic longevity observed for the engineered biofilm system.

**Conclusions:**

Here we provide the first evidence of a recombinant enzyme’s regeneration in an engineered biofilm. The recombinant enzyme was constantly replenished over time as evidenced by dynamic SILAC, which suggests that the engineered *E. coli* biofilms are highly metabolically active, having a not inconsiderable energetic demand. The constant renewal of recombinant enzyme highlights the attractive possibility of utilising this biofilm system as a dynamic platform into which enzymes of interest can be introduced in a “plug-and-play” fashion and potentially be controlled through promoter switching for production of a series of desired fine chemicals.

**Electronic supplementary material:**

The online version of this article (doi:10.1186/s12934-016-0579-3) contains supplementary material, which is available to authorized users.

## Background

The development of novel biocatalysts is crucial in order to provide greener and more sustainable solutions for synthesis of fine chemicals. Enzymatic transformations are becoming increasingly important to the chemical manufacturing and pharmaceutical industries and it is projected that by 2050, in order to move toward greater sustainability and cost reduction, 30 % of chemical syntheses in these sectors will be biocatalytic [1, 2]. One significant challenge to reaching this goal is that many enzymes demonstrate poor stability. Whilst immobilization technologies have been successfully utilised to improve enzyme viability and allow catalyst recycling [3–13], and evolution approaches have been successfully employed to afford enhanced stability [14–25], these approaches must be applied and optimised for each enzyme on a case-by-case basis and can add significant overall cost to the biocatalytic process. Whole cell biocatalysts offer a useful alternative to the use of isolated enzymes, especially for processes requiring the recycling of expensive cofactors [26–37], yet these biocatalysts are not able to operate for prolonged periods of time due to substrate and/or product toxicity and constant exposure to physical and chemical extremes, i.e. shear stress, high temperature, pH variation or solvents. This has driven the need to optimize a given whole-cell process, such as immobilization of cells which provide a more stable environment and the benefit of easy retrieval for reuse [29, 35, 38–45]. In contrast, the naturally immobilized cells or biofilms representing the predominant microbial lifestyle in the natural environment are well known for their resilience [46–51]. Their robustness, which in part is conferred by a well-organized 3-dimensional architecture where cells are embedded and protected by a matrix of secreted extracellular polymeric substances (EPS), allows them to withstand unfavourable conditions that planktonic cells and immobilised enzymes are not able to tolerate [52–56]. These properties lend them to being stable biocatalysts of utility to various industrial sectors: biofilms, mainly multi-species communities of bacteria, are employed in waste water treatment and bioremediation of polluted sites [57–61], and single species biofilms are used for the sustainable production of simple compounds, such as acetic acid, ethanol, butane-2,3-diol and succinic acid [60, 62, 63]. Recently, there is a growing interest in developing and utilising biofilm-based processes, and a new area of utilising engineered single species biofilms for synthesis of desired fine chemicals is beginning to emerge [64–70]. The utilisation of *E. coli* biofilms is attractive due to the wide industrial use of *E. coli* and the ease with which it can be genetically manipulated, e.g., overexpressing genes encoding target enzymes and constructing knockout mutants for control and maximization of target products and elimination of by-products. Previously we have demonstrated how the process of biofilm generation and maturation by *E. coli* K-12 PHL644, which readily forms biofilms due to overproduction of the adhesin curli [71], could be artificially accelerated by simply spinning cells onto glass plates coated with poly-l-lysine [65]. After 6 days maturation of the engineered biofilm, well-organised mushroom-like structures are visible where cells are arranged in multilayers and embedded in the biofilm extracellular matrix. Deep pores and channels developed, conferring a large catalytic surface area to the biofilm [65, 72]. We previously showed that this system could be engineered by constitutive expression of the tryptophan synthase from *Salmonella enterica* (TrpBA) encoded by plasmid pSTB7 [73]. The recombinant TrpBA is composed of two subunits associated as an α_2_β_2_ tetramer, and the β subunit is catalytically independent, mediating the synthesis of l-tryptophan from free indole and l-serine. The engineered biofilm has been employed to catalyse the enantio-selective conversion of haloindoles and l-serine to l-halotryptophans, a series of fine chemicals of value in the synthesis of medicinally relevant compounds [65, 70]. We previously observed that in the biotransformation of 5-chloroindole to 5-chlorotryptophan, strikingly, higher conversions were obtained with the 7 day-old biofilm (conversions of 78 %) than with the immobilised TrpBA enzyme (conversions of 49 %) or planktonic cells expressing recombinant TrpBA (conversions of 40 %) [65]. It was also notable, in our previous experiments, that biotransformation of 5-chloroindole to 5-chlorotryptophan with the engineered biofilm still proceeded at the initial rate following 30 h incubation (Additional file 1: Figure S1a) [65]. Moreover, the biofilm could be reused and, remarkably, there was no observable decrease in catalytic activity after three sequential reaction cycles (Additional file 1: Figure S1b) [65]. Intrigued by the promising catalytic longevity and the potential application of this biofilm system in catalysing a range of required biotransformations, as simple to engineer, plug and play biocatalysts, we sought to investigate the reason for the observed biocatalytic longevity further. Through a pulse-chase SILAC (stable isotope labelling by amino acids in cell cultures) study, we set out to explore the fate of the recombinant enzyme introduced to the biofilm as a catalyst. Our aim was to investigate whether the catalytic longevity of the biofilm is in part due to the regeneration of the recombinant biocatalytic enzyme, beyond the protection conferred by the EPS.

## Results and discussion

### Application of pulse-chase SILAC to follow the fate of the recombinant enzyme expressed in the biofilm

Pulse-chase SILAC experiments involve dynamically following the metabolic behaviour of a cell culture in the presence of an isotopically labelled amino acid, followed by media replacement and the growth of the culture in the presence of the unlabelled amino acid. In this case we wished to explore whether the production of the recombinant enzyme tryptophan synthase was continuous, in engineered biofilms. In a typical SILAC experiment, at given time points, cells are harvested for preparation of protein samples and target proteins are isolated and tracked for label incorporation. Lysine and arginine are the most commonly used labelled amino acids for these studies as they present a trypsin cleavage site (provided they are not followed by a proline residue) and a convenient C terminal label for mass spectrometry (MS) analysis [74, 75]. Other stable isotope labelled amino acids are also frequently used [76] and the choice is usually dictated by the abundance of each amino acid in the protein under investigation. Importantly, the labelled form of the peptides resulting from the trypsin digestion of the parent protein must have a distinct mass, compared to the unlabelled peptide pool in order to be detected in the MS analysis. The optimal additional mass conferred by an isotopically labelled amino acid is plus 4 Da, when exploring expression profiles of a complex series of different proteins or an entire proteome [74]. However, when exploring a single protein, the additional mass of plus 2 Da per amino acid incorporated is more than sufficient to enable ready detection of incorporation, as, for a doubly charged peptide fragment, the incorporation of such a label would result in an increase of 1 Da in the *m/z.* Conventional SILAC analysis involves a single pulse of one isotopically labelled amino acid followed by a single chase [76–78]. However, to gain a greater understanding of our biofilm and whether it regenerates its recombinant biocatalytic enzyme over the course of its maturation, we designed a multistep labelling regiment using a series of two labelled and unlabelled amino acids. To ascertain which two labelled amino acids should be utilised in the SILAC experiment, the amino acid composition and the tryptic digest of the β subunit of TrpBA were evaluated in silico (summarised in Additional file 1: Tables S1, S2, respectively). In TrpBA, lysine and phenylalanine are abundant, at around 5 and 3 % of all amino acids. Of the unique tryptic peptides with five or more amino acids, 71 % contain lysine and 33 % contain phenylalanine. Due to the observation of a high frequency of occurrence of lysine in the tryptic peptides of TrpBA, we therefore chose to use [4,4,5,5-^2^H_4_]-l-lysine as the SILAC amino acid. We chose [2,6-^2^H_2_]-l-phenylalanine as the second labelled amino acid due to our ability to generate the isotopic phenylalanine in plentiful supply, in a cost effective manner, in house. Growing the biofilm in the presence of each of these labels would provide an anticipated shift of 2 *m/z* unit per each [4,4,5,5-^2^H_4_]-l-lysine residue incorporated and a shift of 1 *m/z* unit per each [2,6-^2^H_2_]-l-phenylalanine residue.

**Fig. 1:**
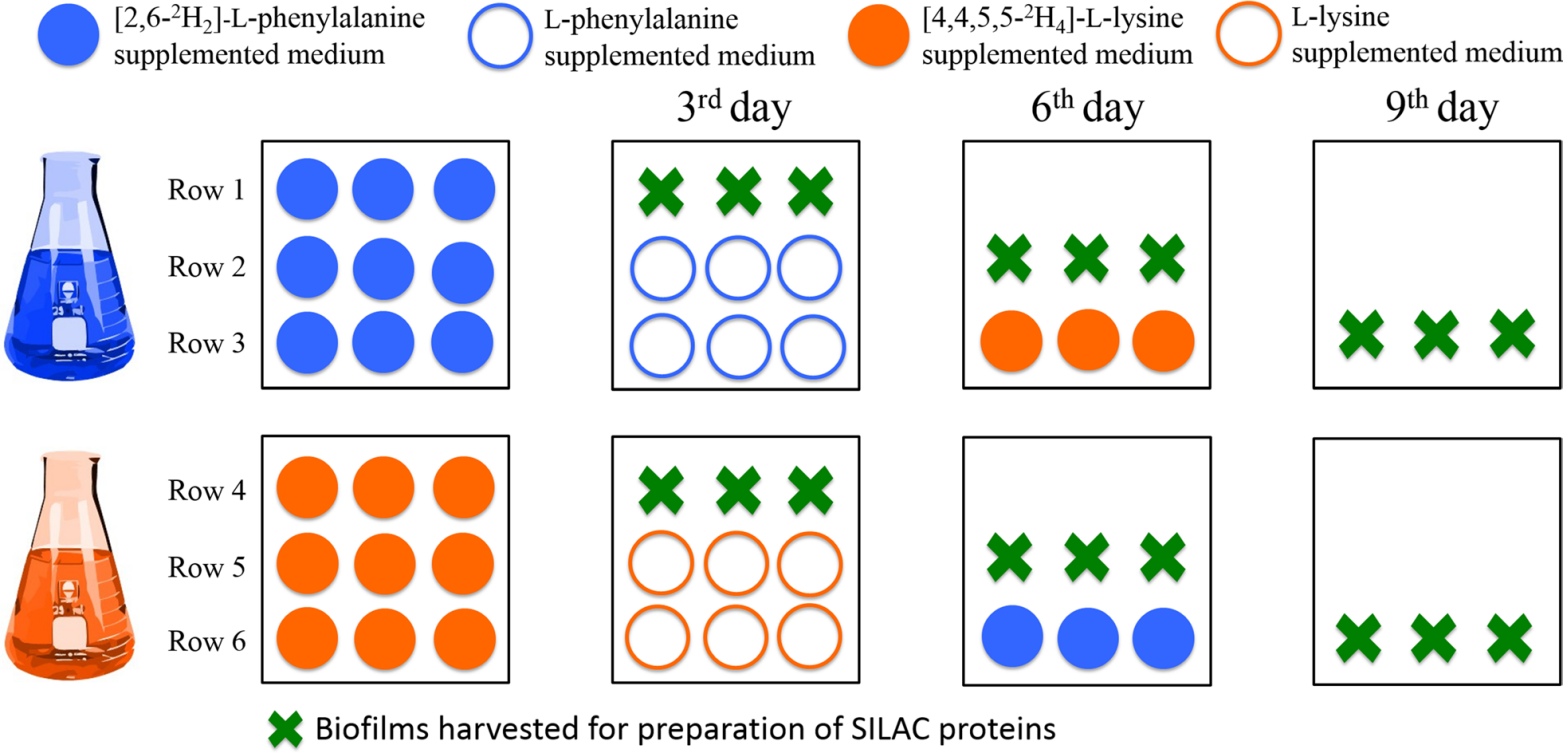
Workflow for the pulse-chase SILAC experiment. For biofilm generation, *E. coli* PHL644 cells in stationary phase were spun down onto poly-l-lysine coated slides placed in 9-deep-well plates. Triplicate sets of biofilms were initially matured in minimal M63 media supplemented with either [2,6-^2^H_2_]-l-phenylalanine (*rows* 1-3, *filled blue circles*) or [4,4,5,5-^2^H_4_]-l-lysine (*rows* 4–6, *filled orange circles*). After 3 days of maturation, biofilms from* rows* 1, 4 were harvested (as denoted by the *crosses*) to prepare protein samples. Labelled media were replaced with fresh unlabelled media (*empty blue circles* for l-phenylalanine and *empty orange circles* for l-lysine) for the chase phase. Following a further 3 days of incubation, the biofilms in* rows* 2, 5 that had matured for 6 days were harvested to extract proteins. The media of the remaining biofilms was again exchanged; fresh media containing [2,6-^2^H_2_]-l-phenylalanine were provided to the biofilms that had been previously supplemented with lysine, and conversely fresh labelled media supplemented with [4,4,5,5-^2^H_4_]-l-lysine were provided to the biofilms that had previously been supplemented with phenylalanine. At day 9, biofilms from rows 3 and 6 were harvested

To provide statistically robust results, in order to explore definitively whether the recombinant enzyme in the biofilm was regenerated, six series of pulse-chase experiments were carried out, in triplicate, as summarised in Fig. 1 (full details provided in the Methods section). Our labelling regimen involved supplementation of sets of individual cultures of the *E. coli* cells, to be used in biofilm formation, each with one of the two isotopically labelled amino acids. After the cultures had grown in the presence of the label, these cells were spin-coated onto glass slides that were then placed within deep-well plates, in the same manner as we have previously reported for the promotion of rapid biofilm formation [65]. Fresh media containing the same labelled amino acid (either labelled lysine or phenylalanine), as the cultures had been initiated in, was reintroduced to each well, and then incubated for 3 days whilst the biofilms started to mature. After 3 days the medium was removed and replaced with fresh media supplemented now with the unlabelled variant of the initial amino acid that had been used for each culture/biofilm i.e. unlabelled phenylalanine (Fig. 1, rows 2, 3) or unlabelled lysine (Fig. 1, rows 5, 6). The biofilms from both rows 1 and 4 were harvested for preparation of target proteins subjected to tryptic digestion followed by liquid chromatography-mass spectrometry (LC–MS) analysis (as detailed in the ‘‘Methods’’ section). After a further 3 days of maturation, biofilms in rows 2 and 5 were harvested and processed for downstream analysis. Again, the media were removed from all of the other biofilms, and the isotopic labelling regimen was swapped with the biofilm in row 3, that had previously been cultured in the presence firstly of deuterated then unlabelled phenylalanine now being supplemented with labelled lysine. Conversely, the biofilm in row 6 that had previously being supplemented firstly with labelled then unlabelled lysine, now being supplemented with labelled phenylalanine. The incubation continued until day 9 with biofilms in rows 3 and 6 being harvested at this stage.

### Identifying and quantifying peptides from SILAC samples reveals the replenishment of the recombinant biocatalytic enzyme within the biofilm

As described above, and in Fig. 1, we designed a labelling regime which included pulsing with labelled amino acids, followed by a chase phase in which unlabelled amino acids, then an alternative labelled amino acid was administered to maturing biofilms. At each time point, where the label was changed (as illustrated in Fig. 1) biofilms were harvested for preparation of target proteins subjected to tryptic digestion, and the tryptic peptides were submitted for LC–MS analyses. The MS data were analysed using the Mascot algorithm (Matrix Science, London, UK) [79] with the modified masses of SILAC amino acids, i.e., [2,6-^2^H_2_]-l-phenylalanine and [4,4,5,5-^2^H_4_]-l-lysine, added as variable modifications in the searches against an internal database at the BSRC (Biological Science Research Complex, University of St Andrews, UK). The data were also searched against the National Centre for Biotechnology Information (NCBI) database [80]. The fragments of the β subunit of TrpBA identified by Mascot, based on at least five biofilm samples, are listed in Additional file 1: Table S3. In total, the identified fragments covered 53 % of all amino acids of TrpBA.

The aim of pulse-chase SILAC experiment performed in this study is to evaluate whether the recombinant enzyme expressed in the biofilm is replenished over time; if the system is static and the catalytic longevity is simply due to the protection of the initial enzyme population, little change or no incorporation in the label would be expected for target proteins coming from biofilms after 6 days of maturation. If the engineered biofilm enables catalytic longevity by renewal of the enzyme and the system is highly dynamic we would anticipate an observation of a change in the incorporation level of the respective labelled amino acid, at 6 days of maturation and beyond. After peptide identification, the MS spectra and ion chromatograms of two tryptic peptides (VGIYFGMK and DPEFQAQFADLLK) from the β subunit of TrpBA were extracted and examined at the MS level to quantify the incorporation level of each SILAC amino acid (the distribution of two peptides along the protein sequence is depicted in Additional file 1: Figure S2). The signal intensities (areas under the curves) from light and heavy peptides provide a quantitative comparison of their relative abundances [75, 78, 81]. The MS spectra of two reference peptides, which each gave nearly identical quantification results, are provided in Additional file 1: Figures S3, S4.

We examined the incorporation level of SILAC amino acid into target proteins from biofilm samples harvested at each time point (day 3, day 6 and day 9). The level of incorporation was quantified by calculating the median of the labelled (heavy, H)/unlabelled (light, L) ratios according the accepted procedure [78]. In accordance with this standard literature procedure for determination of the median of H/L peptide ratios, the peak areas of the extracted ion chromatograms (XICs) of the labelled peptides were measured against the unlabelled peptides. The degree of labelling on target protein was quantified with two reference peptides, as listed above (see Additional file 1: Figures S5, S6 for the XICs of the two peptides). Labelling analysis of the recombinant tryptophan synthase produced by 3 day-old biofilms showed isotopic incorporation levels of ~93 % for biofilms that had been cultured in minimal M63 medium supplemented with [2,6-^2^H_2_]-l-phenylalanine (Fig. 1, row 1 samples), and isotopic incorporation levels of about 38 % for biofilms that had matured in [4,4,5,5-^2^H_4_]-l-lysine supplemented medium (Fig. 1, row 4 samples). The lower incorporation level of deuterated l-lysine was observed, even though supplemented at ten-fold higher concentration, potentially due to the direction of this amino acid towards alternative metabolic pathways, and highlights the benefits of utilising a pair of labelled amino acids.

**Fig. 2:**
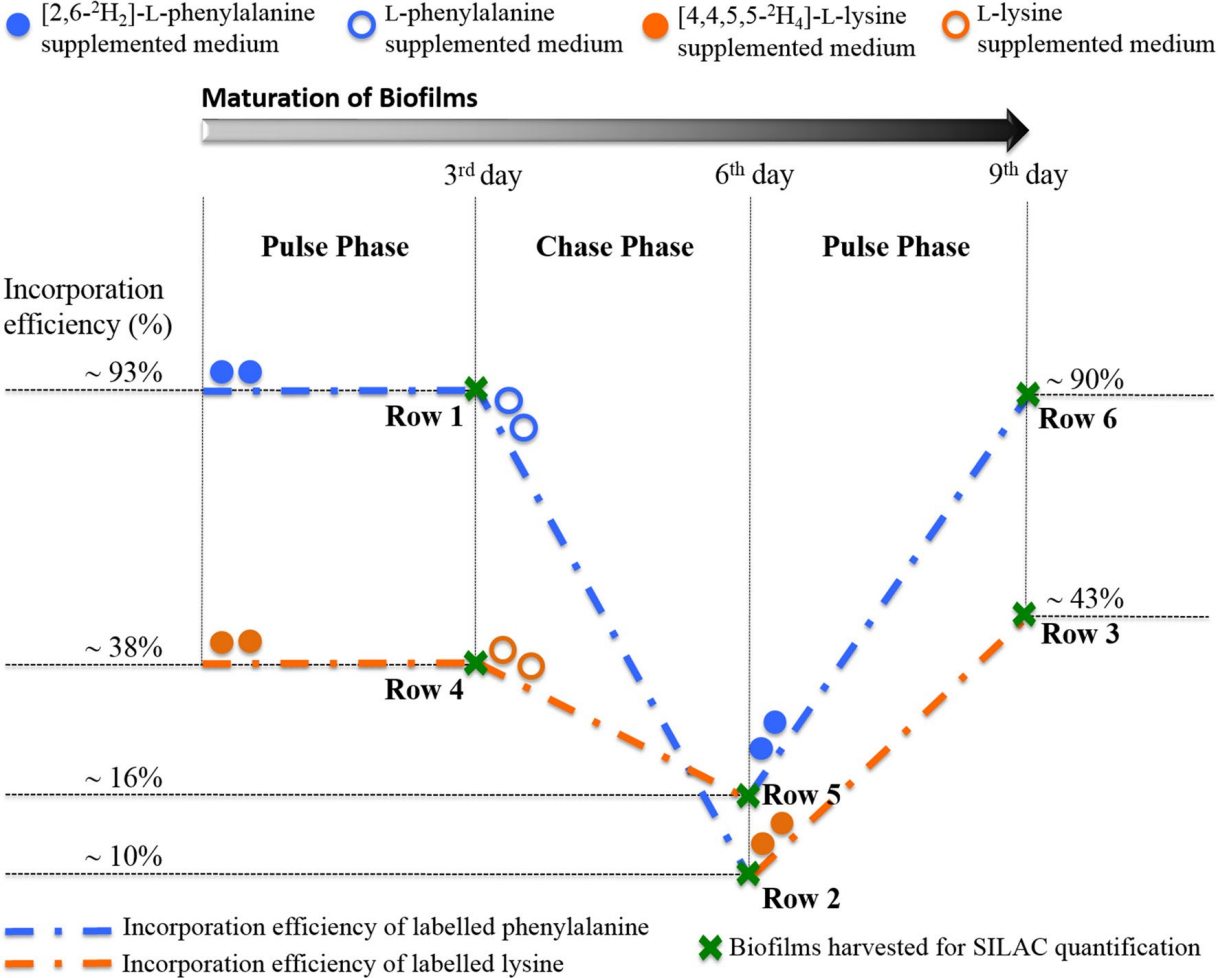
Summary of changes in the incorporation level of SILAC amino acids into target protein. The labelling efficiency is calculated based on the XICs of the two peptides, VGIYFGMK and DPEFQAQFADLLK, using the formula: Incorporation % = [H/L]median/(1 + [H/L]median) ×100. The heavy/light (H/L) SILAC ratios are determined by analysing the ion chromatograms of the heavy and light peptides as they elute from a C18* column*, and then calculating the ratio of the areas under XICs curves. The two reference peptides were detected in the tryptic digest of the recombinant enzyme extracted from 3 day-old biofilms pulsed with deuterated l-phenylalanine (*row 1*) or with deuterated l-lysine (*row 4*), 6 day-old biofilms chased with unlabelled phenylalanine (*row 2*) or with unlabelled lysine (*row 5*), and 9 day-old biofilms cross pulsed with deuterated l-lysine (*row 3*) or with deuterated l-phenylalanine (*row 6*)

To evaluate whether fresh enzymes were produced in both sets of samples, as described above, after 3 days maturation of the engineered biofilm we started the chasing by replacing the labelled medium with fresh medium supplemented with the respective unlabelled amino acids (Fig. 1, rows 2, 3 for phenylalanine and rows 5, 6 for lysine). After 3 more days of maturation, biofilms from rows 2 and 5 chased with unlabelled phenylalanine and lysine respectively, were harvested for preparation of target proteins. As seen in Fig. 2, for the row 2 samples, it was observed that the incorporation level of deuterated l-phenylalanine had dropped from 93 to 10 %; for the row 5 samples, the incorporation level of [4,4,5,5-^2^H_4_]-l-lysine was also observed to decrease from 38 to 16 %. The reduced level of SILAC amino acids observed for three reference peptides suggests that new recombinant enzyme was dynamically generated de novo. We also examined the MS spectra of two reference peptides, detected from the tryptic digest of recombinant enzyme coming from the biofilms chased with unlabelled phenylalanine (Additional file 1: Figures S3, S4; row 2) or unlabelled lysine (Additional file 1: Figures S3, S4; row 5). A drop in the incorporation level of the stable isotope labelled amino acids was observed as indicated by the ratios in the MS spectra of the correlated heavy and light peptides, which is consistent with the quantification analysis based on XICs.

To extend our studies on the enzyme regeneration we sought to explore whether a further change in labelling regiment, past day 6, would continue to differentially label the tryptic peptides. At day 6, biofilm that had previously been administered l-phenylalanine was now pulsed with 300 μM of [4,4,5,5-^2^H_4_]-l-lysine (Fig. 1, row 3) and conversely the biofilm that had been administered l-lysine was now pulsed with [2,6-^2^H_2_]-l-phenylalanine (Fig. 1, rows 6). After a further 3 days of maturation, the biofilms were harvested and processed for isolation of target proteins. Incorporation of labelled lysine into the recombinant tryptophan synthase was observed for the biofilms initially matured in medium containing phenylalanine and vice versa (see Additional file 1: Figures S3, S4 for incorporation of [4,4,5,5-^2^H_4_]-l-lysine and [2,6-^2^H_2_]-l-phenylalanine). Strikingly, incorporation of labelled phenylalanine is observed at ~90 % for biofilm samples in row 6, close to the incorporation level observed for the 3 day-old biofilms in row 1. Conversely, and in agreement with this result, the 9 day-old biofilms in row 3 that had been switched to the labelled lysine pulse now showed an incorporation level of labelled lysine at ~43 %, very similar to the incorporation level of ~38 % observed for the 3 day-old biofilms in row 4. This consistency in labelling level reveals that the recombinant enzyme within the biofilm continues to be completely replenished without perceivable difference in regeneration rates between the 3 day-old and 9 day-old biofilms. For our assessment of the ability of the biofilm samples to catalyse biotransformation, slides containing biofilms that had matured for 7 and 9 days were transferred to the reaction buffer supplemented with 5-chloroindole and incubated at 28 °C for 24 h in an orbital shaker incubator, as detailed in the Methods section. No significant difference was observed in terms of overall percentage chlorotryptophan yield between biotransformations with 7 and 9 days-old biofilms (54 vs 59 %; Additional file 1: Figures S7, S8), confirming that the generated recombinant tryptophan synthases in biofilms are catalytically active. Overall, these findings demonstrate that a key contributor to the striking and useful longevity of the biocatalytic biofilm is the regeneration of the active enzyme.

## Conclusions

The urgent demand for the green, sustainable and cost-effective generation of fine chemicals continues to drive the need for new and effective tools for biotransformations. It is predicted that in the near future almost one-third of industrial chemical synthesis will be mediated by enzymes. To embrace this challenge, not only must new enzymes be discovered and developed, but robust and generic approaches toward the immobilisation, stabilisation and protection of the biocatalyst must be established. Readily engineered, single species biofilms represent an exciting opportunity as biocatalytic platforms into which genes encoding enzymes that catalyse valuable biotransformations can be introduced. The biofilm thereby produces, immobilises and protects the biocatalyst. We envisage the possibility of utilising this biofilm as a cell factory in which enzymes of interest can be introduced into this platform in a “plug and play” fashion for the generation of desired fine chemicals. We had previously observed the biofilm possesses striking biocatalytic longevity, this, we had postulated, was likely to be predominantly due to the protection by the EPS. Our studies of the lifespan and regeneration of the recombinant enzyme, using SILAC, demonstrate that the recombinant enzyme is being constantly regenerated in the matured biofilm, which we believe is fundamental to the longevity of its catalytic activity. Remarkably, we see similar levels of fresh label incorporation in a 9 day-old biofilm as in a 3 day-old biofilm indicating a similar generation level of the recombinant enzyme in the developed biofilm as in the newly formed biofilm. This result is surprising and exciting as it opens the potential, through the careful choice and application of promoters, to switch the biocatalytic function of a mature biofilm, and the products that it generates.

## Methods

### Chemicals, plasmids, bacterial strains and growth conditions

Chemicals used in this study were purchased from Sigma-Aldrich (St Louis, MO, USA), Fisher Scientific (Leicestershire, UK) and Apollo Scientific (Stockport, UK), unless stated otherwise. Solvents were purchased from Sigma-Aldrich and Fisher Scientific. *E. coli* K-12 strain PHL644 (MC4100 *malA*-*kan ompR234*) [71] transformed with pSTB7 plasmid containing *S. enterica trpA* and *trpB* genes and encoding ampicillin resistance [73] is employed in this study.

General microbiological work was performed in a sterile environment using either a laminar flow hood or working around a bunsen flame. A 2 % Distel solution was used to sterilise surfaces prior to work. Strains were routinely grown in LB (Luria–Bertani) broth (10 g L^−1^ NaCl, 10 g L^−1^ tryptone, 5 g L^−1^ yeast extract, pH 7.0) or agar plates supplemented with ampicillin at a final concentration of 100 μg mL^−1^. For solid medium, agar was added at a final concentration of 15 g L^−1^. For long term storage at −80 °C, LB broth containing 20 % of glycerol was used.

For biofilm maturation, M63 medium (100 mM KH_2_PO_4_, 15 mM (NH_4_)_2_SO_4_, 0.8 mM MgSO_4_.7H_2_O, 9 mM FeSO_4_.2H_2_O, 17 mM K-succinate, 10 mM glucose, adjusted to pH 7.0 and supplemented with 100 μg mL^−1^ ampicillin) was used as it has been shown to promote curli production. For SILAC experiment, M63 medium was supplemented with either [4,4,5,5-^2^H_4_]-l-lysine (Sigma-Aldrich, St Louis, MO, USA) or in house [2,6-^2^H_2_]-l-phenylalanine.

### Pulse-chase SILAC experiments

SILAC was explored as a tool to determine whether or not the tryptophan synthase was constantly regenerated over time within the biofilm. We set pulse-chase experiments in which biofilms were matured in M63 medium supplemented with stable isotope labelled amino acids (pulse phase) and subsequently with unlabelled amino acids (chase phase). [4,4,5,5-^2^H_4_]-l-lysine (Sigma-Aldrich) and in house [2,6-^2^H_2_]-l-phenylalanine were used during the pulse phases and supplemented to the medium at final concentrations of 300 and 30 μM, respectively. The same concentrations were used for unlabelled l-phenylalanine and l-lysine during the chase phases.

Biofilms were grown by employing a spin coating method as previously described [65] with slight modification. 75 × 25 mm microscope slides (VWR International, Leicestershire, UK) were cut into 25 × 25 mm squares, sterilized and coated with approximately 1.5 mL of 0.1 % w/v poly-l-lysine in water (Sigma-Aldrich). Slides were dried in an oven at 60 °C and then transferred into 9-deep-well plates. A culture of PHL644 pSTB7 [71] was streaked onto a LB agar plate with 100 µg/mL of ampicillin and single colonies were inoculated into M63 medium containing ampicillin. When the exponential growth phase (OD_600_ of 0.2) was reached, 600 μL aliquots were inoculated in 60 mL volume of M63 supplemented with either 30 μM of labelled phenylalanine or 300 μM of labelled lysine in 250 mL Erlenmeyer flasks, incubated at 28 °C, 150 rpm, in an incubator with a 19 mm throw until stationary phase (OD_600_ of 2.0) was reached. Cultures were then centrifuged at 1851*g* for 15 min and resuspended in fresh M63 medium supplemented with either 30 μM of labelled phenylalanine (triplicate cultures illustrated in Fig. 1, rows 1–3) or 300 μM of labelled lysine (triplicate cultures illustrated in Fig. 1, rows 4–6) to an OD_600_ ~1.0. 4 mL Aliquots of these suspensions were transferred to each well of a 9-deep-well plate containing a 25 × 25 mm poly-l-lysine coated slide (the labelled phenylalanine containing culture was introduced 9 deep well plates, illustrated in Fig. 1, rows 1–3 and the labelled lysine containing culture introduced into a further 9 deep well plates (Fig. 1, rows 4–6), enabling each experiment to be carried out in triplicate). Cells were deposited onto the slides by centrifuging at 2200*g* for 15 min using a JS 5.3 rotor in an Avanti JXN-26 centrifuge (Beckman, Munich, Germany), media was removed and 4 mL of fresh M63 media supplemented with the same labelled amino acid (either 30 μM of labelled phenylalanine or 300 μM of labelled lysine), as the cells were previously grown in, was added. The biofilm samples were incubated at 28 °C in an orbital shaker incubator at 50 rpm with a throw of 19 mm and left to mature. The 18 biofilms were initially left to mature in the two sets of labelled M63 media for 3 days (pulse phase). At day 3, three of the samples that had been grown in media enriched with labelled phenylalanine and 3 of the samples that had been grown in media enriched with labelled lysine were harvested for analysis (see next section). Media was removed from all of the remaining 12 samples and replaced with 4 mL of fresh media supplemented with either 30 μM of unlabelled phenylalanine (Fig. 1, rows 2, 3) or 300 μM of unlabelled lysine (Fig. 1, rows 5, 6), these samples were returned to incubate at 28 °C in an orbital shaker incubator at 50 rpm with a throw of 19 mm. On day 6, six further samples corresponding to Fig. 1, rows 2, 5 were harvested; the media from the 6 remaining samples removed and replaced with 4 mL fresh media containing the alternative label i.e. the samples that had previously been supplemented with phenylalanine were now supplemented with 300 μM of labelled lysine (Fig. 1, row 3) and those supplemented previously with lysine were now supplemented with 30 μM of labelled phenylalanine (Fig. 1, row 6). The samples were incubated for a further 3 days, under the same conditions prior to their harvesting and analysis.

### Preparation of protein samples for mass spectrometry, LC-MS detection and analysis on the TripleTOF 5600

At each time point (every 3 days), slides containing the biofilms were removed from the deep well plates. Planktonic cells were removed from the biofilm by gentle submersion and washing in aliquots of phosphate buffer (0.1 M KH_2_PO_4_, 7 mM serine, 0.1 mM pyridoxal phosphate (PLP), adjusted to pH 7.0), having an average of optical density (OD_600_) of 0.65 for 3 day-old biofilm, 0.25 for 6 day-old biofilm and 0.31 for 9 day-old biofilm in 4 mL phosphate buffer. Slides were transferred to 50 mL centrifuge tubes, immersed in phosphate buffer and the biofilms were detached by vigorous vortexing. The empty slides were carefully removed and washed and the resultant suspended cells were recovered by centrifuging at 17,000*g*. The biomass was then suspended in lysis buffer (50 mM Tris–HCl, pH 8.0, 25 mM NaCl, 5 % glycerol, 2.5 mM EDTA, 1 mg/mL lysozyme, Sigma) to which 1 volume of 2× SDS loading dye (100 mM Tris–HCl, pH 6.8, 4 % (w/v) SDS, 0.2 % (w/v) bromophenol blue, 20 % (v/v) glycerol, 200 mM DTT) was added; samples were then denatured, loaded in a 4–12 % polyacrylamide gel (NuPage, Thermo Fisher Scientific). After electrophoresis, gels were immersed in staining solution (50 % methanol, 40 % water, 10 % acetic acid, 0.1 % Coomassie Brilliant Blue G-250), followed by destaining. The bands of interest were excised from the gel, cut into 1 mm cubes and subjected to in-gel digestion, using a ProGest Investigator in-gel digestion robot (Genomic Solutions, Ann Arbor, Michigan, USA) according to standard protocols [82]. Briefly, the gel cubes were destained by washing with acetonitrile and subjected to reduction and alkylation before digestion with trypsin at 37 °C. The peptides were extracted with 10 % formic acid and concentrated down to 20 μL using a SpeedVac (Savant™, Thermo Fisher Scientific).

Extracted peptides were separated on an Acclaim PepMap 100 C18 trap and an Acclaim PepMap RSLC C18 column (Thermo Fisher Scientific), using a nanoLC Ultra 2D plus loading pump and nanoLC as-2 autosampler (Eskigent™, Woodlands, Singapore). The peptides were eluted with a gradient of increasing acetonitrile, containing 0.1 % formic acid (5–40 % acetonitrile in 15 min, 40–95 % in a further 1 min, followed by 95 % acetonitrile to clean the column, before reequilibration to 5 % acetonitrile). The eluate was sprayed into a TripleTOF 5600+ electrospray tandem mass spectrometer (Sciex, Massachusetts, USA) and analysed in Information Dependent Acquisition (IDA) mode, performing cycles of 250 ms of MS followed by 100 ms MS analyses on the 15 most intense peaks seen by MS.

### MS data analysis and calculation of SILAC ratios

The MS data file generated via the ‘Create mgf file’ script in PeakView v 2.1 (Sciex, Massachusetts, USA) was analysed using the Mascot algorithm (Matrix Science, London, UK) [79], against the NCBI database [80] with no species restriction, trypsin as the cleavage enzyme and carbamidomethyl as a fixed modification of cysteines and methionine oxidation as a variable modification, to confirm the presence of the *S. enterica* tryptophan synthase TrpBA in the *E. coli* background. The data file was then searched against an internal database which includes the tryptophan synthase sequence from *S. enterica*. Additional variable modifications of [2,6-^2^H_2_]-l-phenylalanine and [4,4,5,5-^2^H_4_]-l-lysine, were added to the search parameters. Extracted ion chromatograms (XICs) corresponding to the peaks of interest, identified by the Mascot search, as peptides from tryptophan synthase were obtained through PeakView software 2.2. The level of incorporation was quantified by calculating the median of the labelled (heavy, H)/unlabelled (light, L) ratios according to the formula: Incorporation % = [H/L]_median_/(1 + [H/L]_median_) × 100 [78]. Ratios of labelled and unlabelled peptides were calculated based on the peak area of XICs (areas under the curves).

### Biofilm-catalysed biotransformation

Biotransformations were carried out as previously described [65, 70] using engineered biofilms that had matured for 7 days and 9 days in a potassium phosphate reaction buffer (0.1 M KH_2_PO_4_, 7 mM Serine, 0.1 mM pyridoxal phosphate (PLP), adjusted to pH 7.0). The biofilm maturation M63 medium was carefully removed from the biofilm covered slide by using a syringe. Unadhered, planktonic cells were removed from the biofilm by gentle resubmersion and washing in aliquots of reaction buffer, which was then also removed. Each of washed biofilm slides removed from the media after 7 and 9 days of maturation was then submerged in 4 mL reaction buffer. To start the biotransformation, the washed slides coated with 7 day-old biofilms (approximately 1.70 mg of total cellular protein present in the biofilms) and 9 day-old biofilms (~1.83 mg of protein) were submerged in 4 mL reaction buffer supplemented with 5 % (v/v) DMSO and 2 mM 5-chloroindole and incubated in an orbital shaker incubator (28 °C, 50 rpm with a 19 mm throw) for 24 h. The total protein content of the biofilm (average of 3 repeats) was determined using a Bradford protein assay and a protocol from Li et al. [83]. 5-chlorotryptophan concentrations in biotransformation samples were analysed using a Waters Acquity UPLC system equipped with a Waters Acquity BEH C18 1.7 µm 2.1 × 50 mm column and PDA detector monitoring absorbance at 280 nm (solvent A: 0.1 % TFA in water, solvent B: acetonitrile. Gradient: 0 min, 5 % B; 0.5 min, 5 % B; 2.0 min, 50 % B; 0.5 mL/min). For analysis, solutions were taken from the biotransformation reaction and centrifuged (16,060*g*, 5 min), and equal volume of methanol was then added. The concentration of 5-chlorotryptophan was calculated based on a standard curve of UPLC peak area versus concentration. Samples of 5-chlorotryptophan of known concentration, ranging from 62.5 to 500 µM, were analysed by UPLC using the method described above. A standard curve of the relationship between UPLC peak area (y) and concentration (x) was constructed and the relationship between peak area and concentration was found to be linear (y = 4866.1x − 596.4). From this standard curve the unknown concentration of 5-chlorotryptophan in the biotransformation samples was determined and an estimation of the overall yield (Y) was calculated (Y % = 5-chlorotryptophan concentration/initial 5-chloroindole concentration). For LC–MS analysis samples dissolved in water: methanol 50:50 were separated using a Dionex Ultimate 3000 RS system (solvent A: 0.1 % FA in water, solvent B: acetonitrile. Gradient: 0 min, 5 % B; 0.5 min, 5 % B; 5 min, 37.5 % B; 5.1 min, 90 % B; 7 min, 90 % B; 0.35 mL/min), ionised using a H-ESI source (positive ion mode, 3.5 kV, heater 325 °C, capillary 350 °C, S-lens RF 47 %, sheath gas 55, auxillary gas 20, sweep gas 2) and analysed using a Thermo Fisher Orbitrap Velos Pro (30,000 resolution, 100–800 *m/z*).

## Additional file

**Additional file 1: Figure S1.** Biofilm-mediated biotransformation (previous work). **Table S1.** Amino acid composition of the subunit β of TrpBA calculated by ProtParam (Expasy). **Table S2** In silico tryptic digest of the subunit β of TrpBA with Peptide Cutter tool (Expasy). **Table S3** Fragments of the subunit β of TrpBA identified by Mascot. **Figure S3.** MS spectra analysis of the peptide VGIYFGMK of TrpBA. **Figure S4.** MS spectra of the peptide DPEFQAQFADLLK of TrpBA. **Figure S5.** The XICs of the reference peptide VGIYFGMK. **Figure S6.** The XICs of the peptide DPEFQAQFADLLK. **Figure S7.** Percentage of 5-chlorotryptophan accumulation, monitored by UPLC. **Figure S8.** LC-MS analysis of formation of 5-chlorotryptophan.

## Abbreviations

SILAC: stable isotopic labelled amino acids in cell cultures
EPS: extracellular polymeric substances
TrpBA: tryptophan synthase from *Salmonella enterica*
MS: mass spectrometry
LC–MS: liquid chromatography–mass spectrometry
XICs: extracted ion chromatograms
PLP: pyridoxal phosphate
