# Supplementary material for: Rapid enzyme regeneration results in the striking catalytic longevity of an engineered, single species, biocatalytic biofilm
Source: Microb Cell Fact. 2016 Oct 21;15:180. doi: 10.1186/s12934-016-0579-3 (PMC5073922; doi:10.1186/s12934-016-0579-3)
Supplement: Supplementary file 1 — Additional file 1: Figure S1. Biofilm-mediated biotransformation (previous work). Table S1. Amino acid composition of the subunit β of TrpBA calculated by ProtParam (Expasy). Table S2 In silico tryptic digest of the subunit β of TrpBA with Peptide Cutter tool (Expasy). Table S3 Fragments of the subunit β of TrpBA identified by Mascot. Figure S3. MS spectra analysis of the peptide VGIYFGMK of TrpBA. Figure S4. MS spectra of the peptide DPEFQAQFADLLK of TrpBA. Figure S5. The XICs of the reference peptide VGIYFGMK. Figure S6. The XICs of the peptide DPEFQAQFADLLK. Figure S7. Percentage of 5-chlorotryptophan accumulation, monitored by UPLC. Figure S8. LC-MS analysis of formation of 5-chlorotryptophan. [file 12934_2016_579_MOESM1_ESM.docx]

**Rapid enzyme regeneration results in the striking catalytic longevity of an engineered, single species, biocatalytic biofilm**

**Supporting Information**

Xiaoxue Tong^a,b^, Tania Triscari Barberi^a,b^, Catherine H. Botting^b^, Sunil V. Sharma^a,b^, Mark J. H. Simmons^c^, Tim W. Overton^c^ and Rebecca J. M. Goss^a,b *^

^a^ School of Chemistry, University of St. Andrews, St. Andrews, UK, KY16 9ST

^b^ Biomedical Sciences Research Complex, University of St. Andrews, St. Andrews, UK, KY16 9ST

^c^ School of Chemical Engineering, University of Birmingham, Edgbaston, Birmingham, UK, B152TT

[xt4@st-andrews.ac.uk](mailto:xt4@st-andrews.ac.uk); [ttb@st-andrews.ac.uk](mailto:ttb@st-andrews.ac.uk); [cb2@st-andrews.ac.uk](mailto:cb2@st-andrews.ac.uk); [svs4@st-andrews.ac.uk](mailto:svs4@st-andrews.ac.uk); [m.j.simmons@bham.ac.uk](mailto:m.j.simmons@bham.ac.uk); [t.w.overton@bham.ac.uk](mailto:t.w.overton@bham.ac.uk); ^*^[rjmg@st-andrews.ac.uk](mailto:rjmg@st-andrews.ac.uk);

**Tables S1-S3 2-4**

Table S1: Amino acid composition of the subunit β of TrpBA calculated by ProtParam (Expasy) 2

Table S2: *In silico* tryptic digest of the subunit β of TrpBA with Peptide Cutter tool (Expasy) 3

Table S3: Fragments of the subunit β of TrpBA identified by Mascot 4

**Figures S1-S8 5-12**

Figure S1: Biofilm-mediated biotransformation (previous work) 5

Figure S2: The amino acid sequence of the subunit β of TrpBA 6

Figure S3: MS spectra analysis of the peptide VGIYFGMK of TrpBA 7

Figure S4: MS spectra of the peptide DPEFQAQFADLLK of TrpBA 8

Figure S5: The XICs of the reference peptide VGIYFGMK 9

Figure S6: The XICs of the peptide DPEFQAQFADLLK 10

Figure S7: Percentage of 5-chlorotryptophan accumulation, monitored by UPLC 11

Figure S8: LC-MS analysis of formation of 5-chlorotryptophan 12

**Reference 13**

**Table S1:** Amino acid composition of the subunit β of TrpBA calculated by ProtParam (Expasy).

| Asn (N) | 2.8% |
| --- | --- |
| Asp (D) | 4.5% |
| Cys (C) | 1.3% |
| Gln (Q) | 4.3% |
| Glu (E) | 7.1% |
| Gly (G) | 11.1% |
| His (H) | 3.5% |
| Ile (I) | 6.0% |
| Leu (L) | 9.6% |
| Lys (K) | 4.8% |
| Met (M) | 3.8% |
| Phe (F) | 3.3% |
| Pro (P) | 4.5% |
| Ser (S) | 5.0% |
| Thr (T) | 5.3% |
| Trp (W) | 0.3% |
| Tyr (Y) | 3.0% |
| Val (V) | 4.8% |

**Table S2:** *In silico* tryptic digest of the subunit β of TrpBA with Peptide Cutter tool (Expasy).

| **Subunit β** | | | | |
| --- | --- | --- | --- | --- |
| **Position of cleavage site** | **Resulting peptide sequence** | **Peptide length [aa]** | **Peptide mass [Da]** | **Cleavage probability** |
| 37 | MTTLLNPYFGEFGGMYVPQILMPALNQLEEAFVSAQK | 37 | 4149.847 | 100 % |
| 50 | DPEFQAQFADLLK | 13 | 1521.690 | 100 % |
| 55 | NYAGR | 5 | 579.613 | 74.6 % |
| 61 | PTALTK | 6 | 629.754 | 100 % |
| 70 | CQNITAGTR | 9 | 963.076 | 100 % |
| 76 | TTLYLK | 6 | 737.894 | 91.8 % |
| 77 | R | 1 | 174.203 | 100 % |
| 87 | EDLLHGGAHK | 10 | 1076.177 | 100 % |
| 99 | TNQVLGQALLAK | 12 | 1255.480 | 87.3 % |
| 100 | R | 1 | 174.203 | 100 % |
| 103 | MGK | 3 | 334.434 | 90.7 % |
| 129 | SEIIAETGAGQHGVASALASALLGLK | 26 | 2464.801 | 100 % |
| 131 | CR | 2 | 277.342 | 100 % |
| 137 | IYMGAK | 6 | 681.848 | 90.6 % |
| 141 | DVER | 4 | 517.539 | 100 % |
| 148 | QSPNVFR | 7 | 846.941 | 100 % |
| 150 | MR | 2 | 305.395 | 100 % |
| 167 | LMGAEVIPVHSGSATLK | 17 | 1710.022 | 86.4 % |
| 175 | DACNEALR | 8 | 890.966 | 91.8 % |
| 202 | DWSGSYETAHYMLGTAAGPHPYPTIVR | 27 | 2978.288 | 100 % |
| 206 | EFQR | 4 | 578.626 | 100 % |
| 213 | MIGEETK | 7 | 806.929 | 100 % |
| 219 | AQILDK | 6 | 686.806 | 83.8 % |
| 222 | EGR | 3 | 360.370 | 100 % |
| 272 | LPDAVIACVGGGSNAIGMFADFINDTSVGLIGVEPGGHGIETGEHGAPLK | 50 | 4862.463 | 100 % |
| 275 | HGR | 3 | 368.396 | 100 % |
| 283 | VGIYFGMK | 8 | 914.130 | 91.2 % |
| 321 | APMMQTADGQIEESYSISAGLDFPSVGPQHAYLNSIGR | 38 | 4039.460 | 100 % |
| 337 | ADYVSITDDEALEAFK | 16 | 1786.910 | 100 % |
| 341 | TLCR | 4 | 491.606 | 100 % |
| 360 | HEGIIPALESSHALAHALK | 19 | 1994.281 | 100 % |
| 363 | MMR | 3 | 436.588 | 100 % |
| 368 | EQPEK | 5 | 629.668 | 79.9 % |
| 379 | EQLLVVNLSGR | 11 | 1227.426 | 100 % |
| 382 | GDK | 3 | 318.330 | 37.7 % |
| 392 | DIFTVHDILK | 10 | 1200.400 | 100 % |
| 394 | AR | 2 | 245.282 | 100 % |
| 397 | GEI | 3 | 317.342 | - |

**Table S3**: Fragments of the subunit β of TrpBA identified by Mascot

| Light (m/z) | Heavy (m/z) | Sequence |
| --- | --- | --- |
| 761.38 | 763.39/1 Lys  763.39/2 Phe | DPEFQAQFADLLK |
| 596.32 | 598.33/1 Lys | NYAGRPTALTK |
| 447.77  411.55 | 449.78/1 Lys  412.89/1 Lys | TTLYLKR  REDLLHGGAHK |
| 628.37 | 630.38/1 Lys | TNQVLGQALLAK |
| 822.12 | - | SEIIAETGAGQHGVASALASALLGLK |
| 424.22 | 425.23/1 Phe | QSPNVFR |
| 570.64 | 571.98/1 Lys | LMGAEVIPVHSGSATLK |
| 782.16  745.10 | -  - | DACNEALRDWSGSYETAHYMLGTAAGPHPYPTIVR  DWSGSYETAHYMLGTAAGPHPYPTIVR |
| 515.28  457.75  893.92  665.36  920.00  614.35 | 517.30/1 Lys  459.75/1 Lys  458.75/1 Phe  895.93/1 Lys  894.92/1 Phe  666.71/1 Lys  -  - | AQILDKEGR  VGIYFGMK  ADYVSITDDEALEAFK  HEGIIPALESSHALAHALK  EQPEKEQLLVVNLSGR  EQLLVVNLSGR |

Note: In total, the fragments, detected in at least five of the six biofilm samples, covered 53% of the total protein sequence.


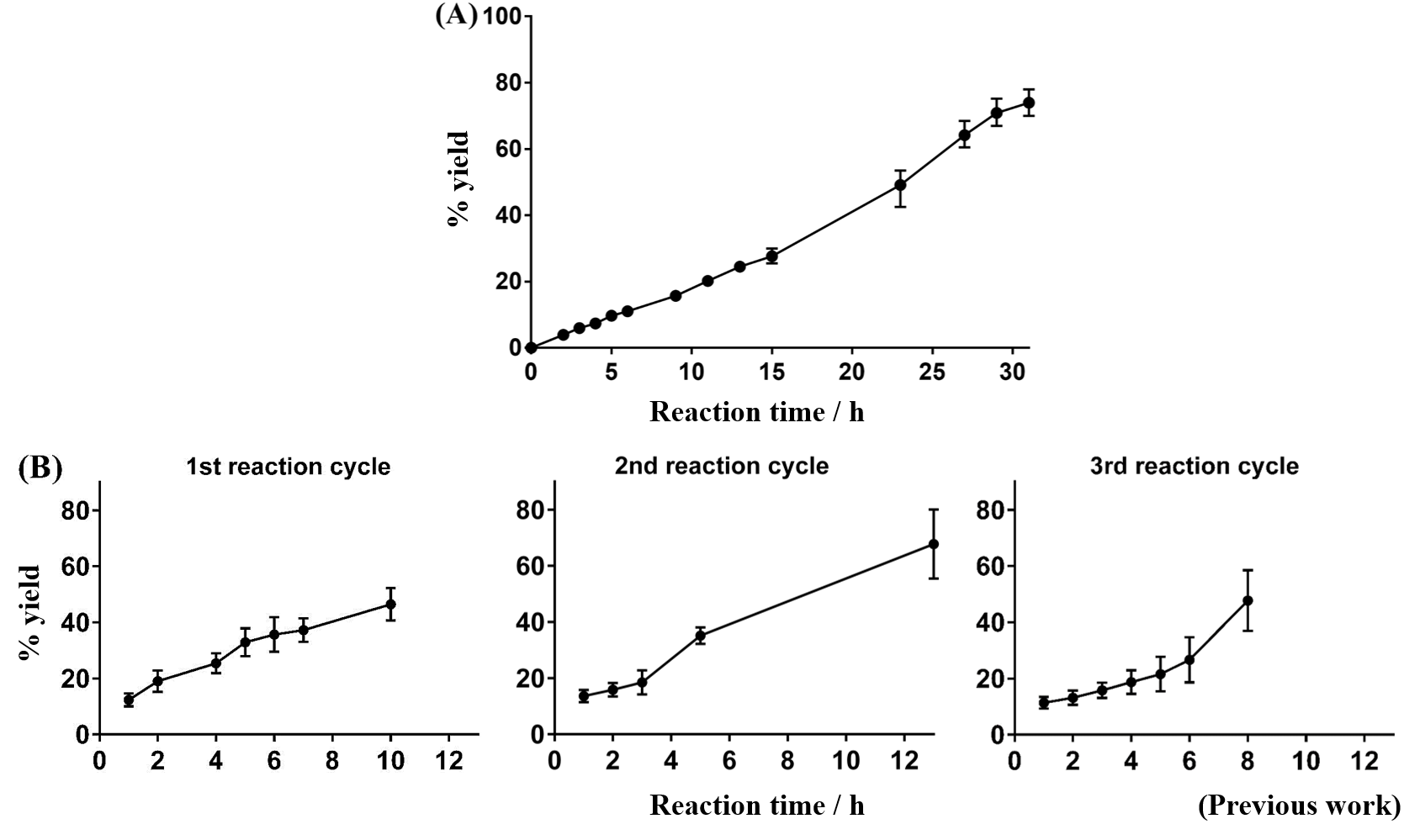


**Figure S1:** Biofilm-mediated biotransformation (previous work). A. Percentage of 5-chlorotryptophan accumulation during biotransformation of 5-chloroindole performed with the engineered *E.coli* biofilm that had matured for 7 days plotted against time. B. Three sequential 5-chloroindole biotransformation reactions with the same biofilm. Recycling the biofilm leads to little observed loss in activity. Data are mean and s.d. [1].


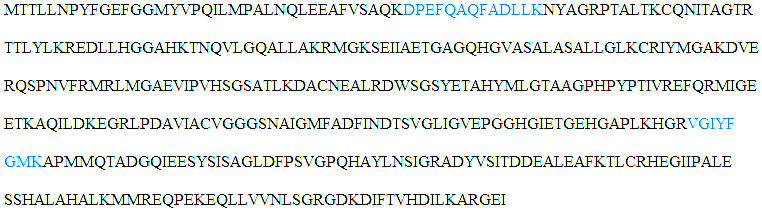


**Figure S2:** The amino acid sequence of the subunit β of TrpBA. The two tryptic peptides identified by LC-MS used for the calculation of labelling efficiency is coloured in blue.


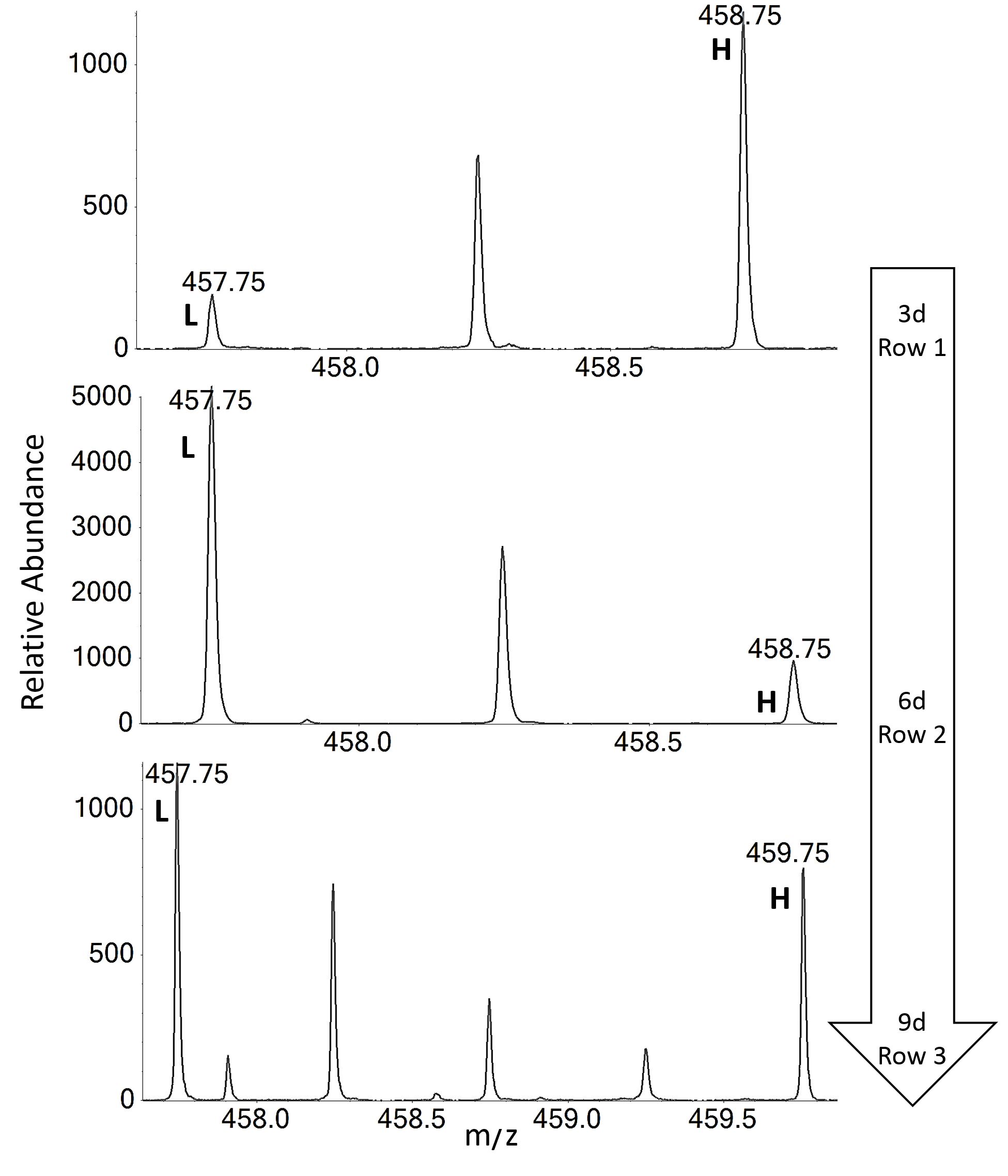

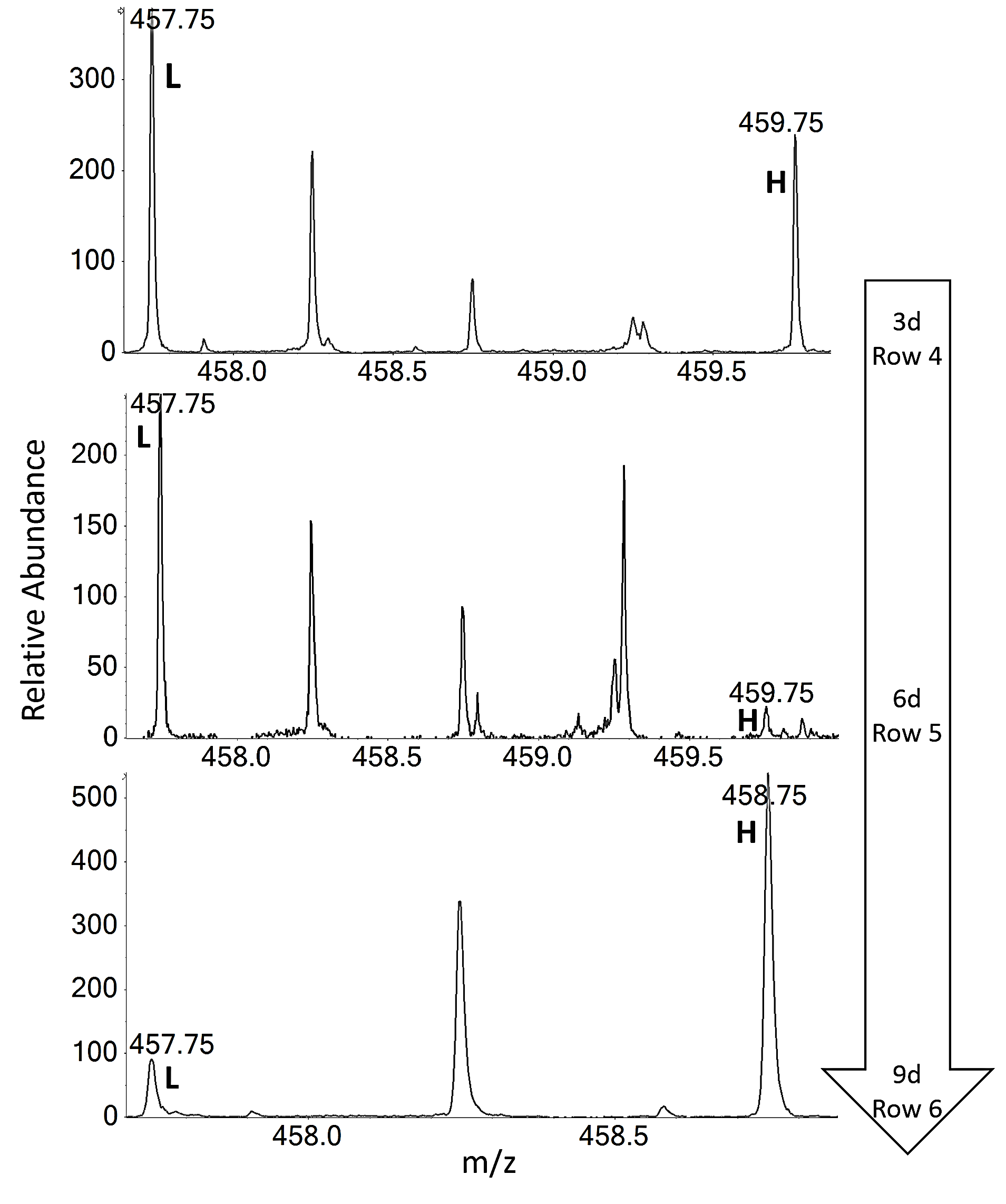


**Figure S3:** MS spectra analysis of the peptide VGIYFGMK detected in the tryptic digest of the recombinant protein coming from 3-day-old biofilms pulsed with deuterated L-phenylalanine (row 1) or with deuterated L-lysine (row 4), 6-day-old biofilms chased with unlabelled phenylalanine (row 2) or with unlabelled lysine (row 5), and 9-day-old biofilms cross pulsed with deuterated L-lysine (row 3) or with deuterated L-phenylalanine (row 6). The peaks at ~457.75 m/z annotated with L are peaks of the light peptide VGIYFGMK. The peaks at ~458.75 m/z and at ~459.75 m/z, both annotated with H, correspond to the peptide containing a labelled phenylalanine residue and a labelled lysine residue, respectively. Incorporation level of isotopically labelled amino acid is evaluated based on the ratios in the MS spectrum of the correlated heavy and light peptides.


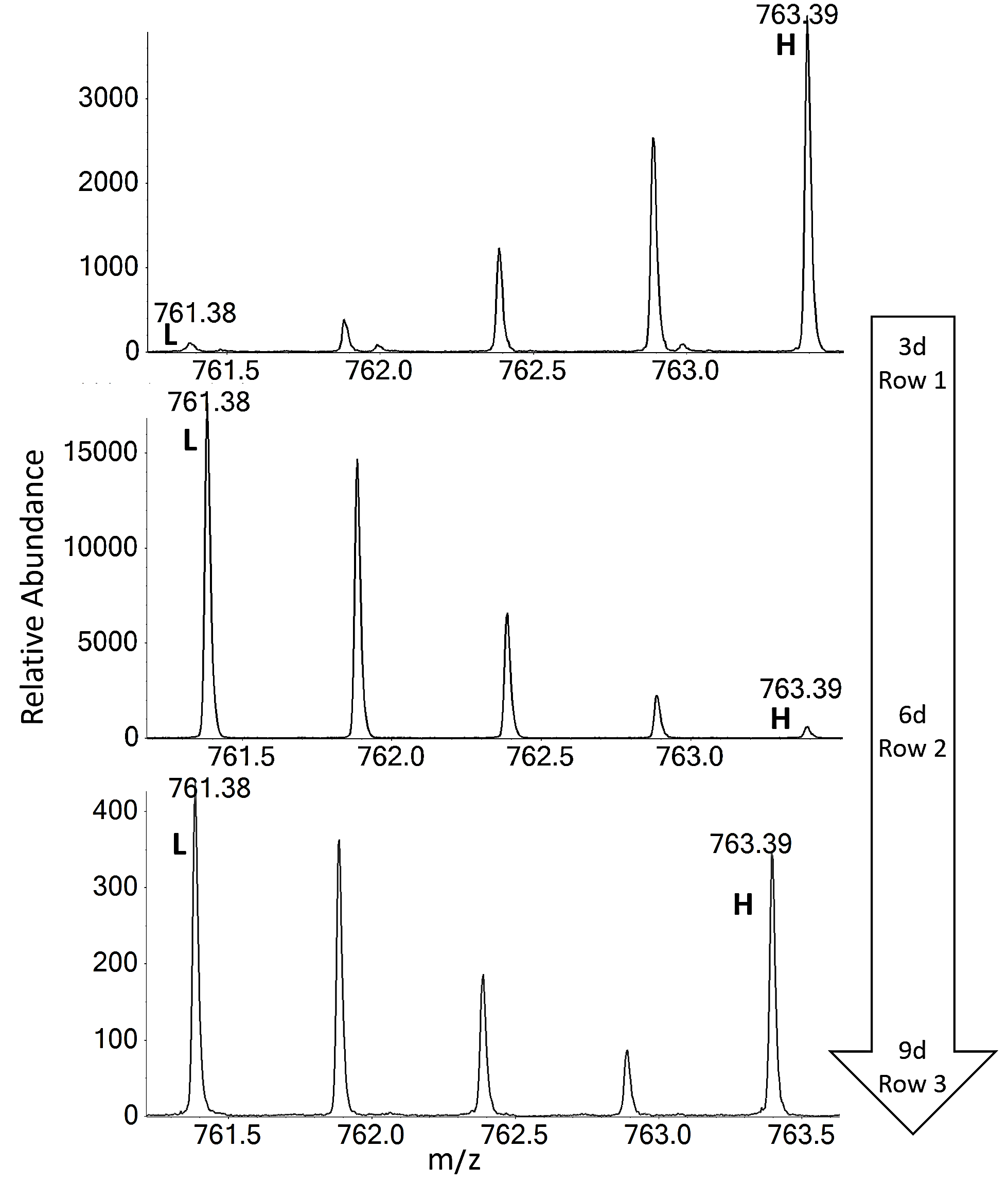

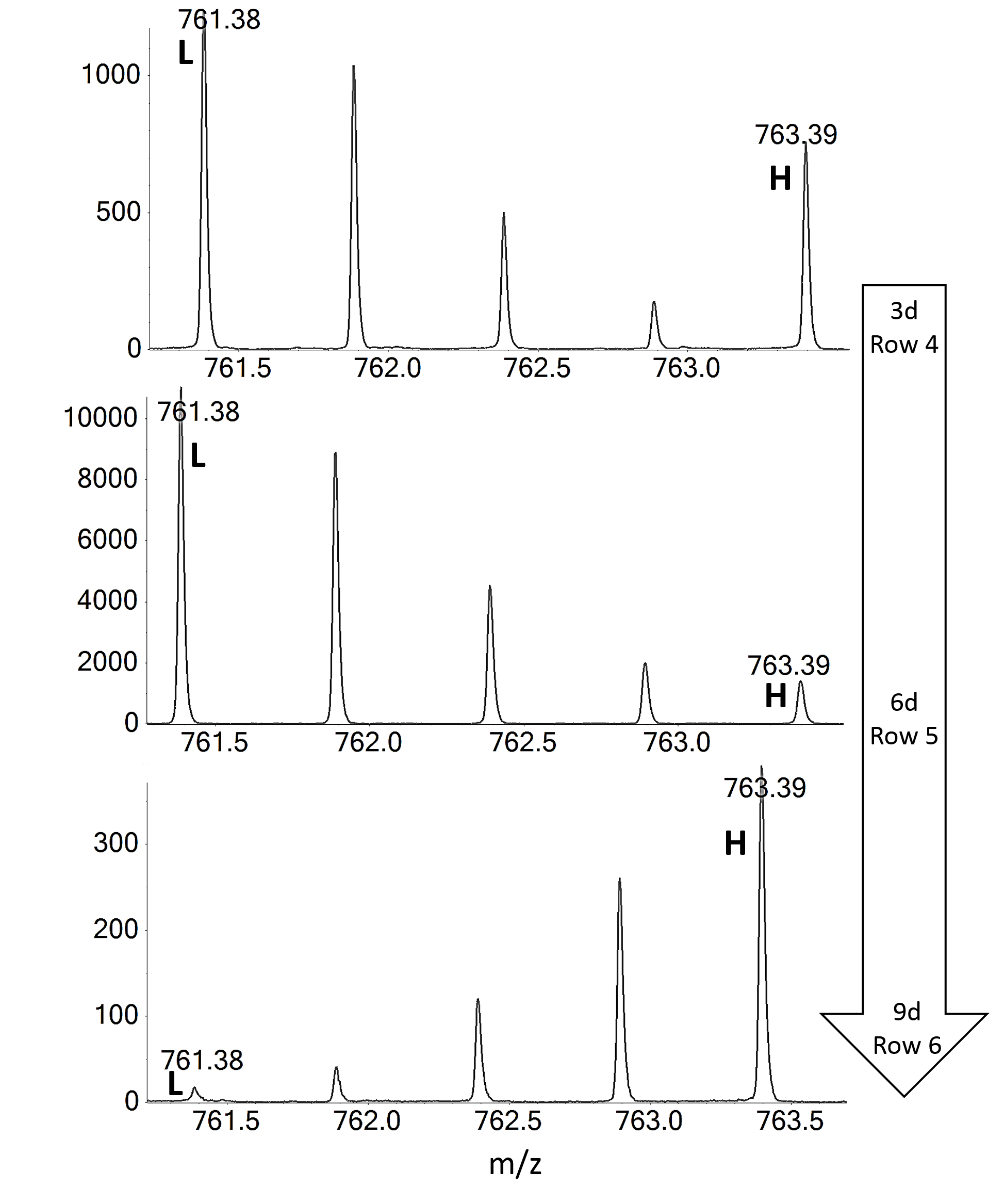


**Figure S4:** MS spectra of the peptide DPEFQAQFADLLK acquired after the tryptic digest of the recombinant protein resulting from 3-day-old biofilms pulsed with deuterated L-phenylalanine (row 1) or with deuterated L-lysine (row 4), 6-day-old biofilms chased with unlabelled phenylalanine (row 2) or with unlabelled lysine (row 5), and 9-day-old biofilms cross pulsed with deuterated L-lysine (row 3) or with deuterated L-phenylalanine (row 6). The peaks at ~761.38 m/z annotated with L are peaks of the light peptide DPEFQAQFADLLK. The peaks at ~763.39 m/z annotated with H correspond to the peptide containing two labelled phenylalanine residues or the peptide with one labelled lysine residue. Incorporation level of isotopically labelled amino acid is evaluated based on the ratios in the MS spectrum of the correlated heavy and light peptides.


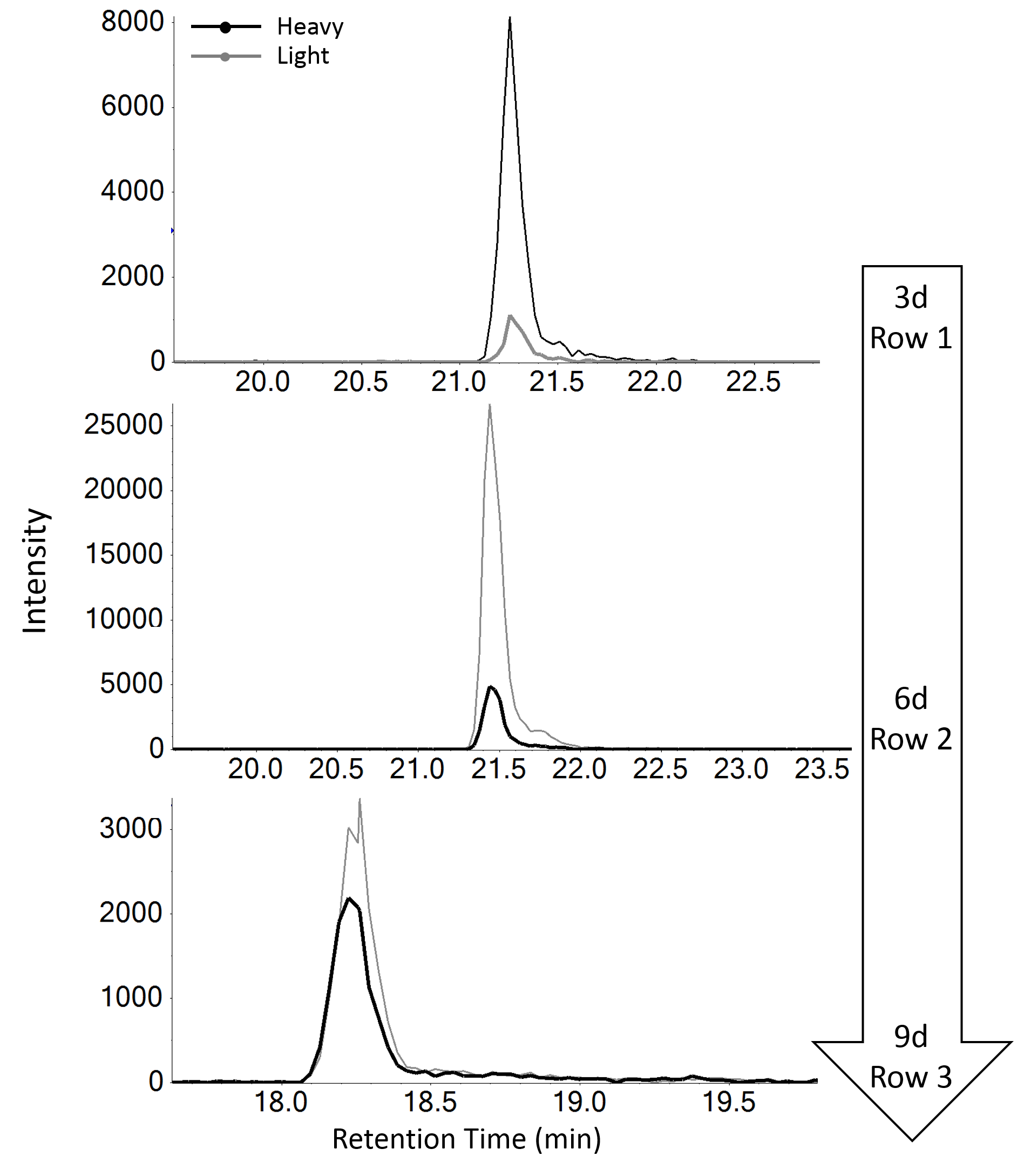

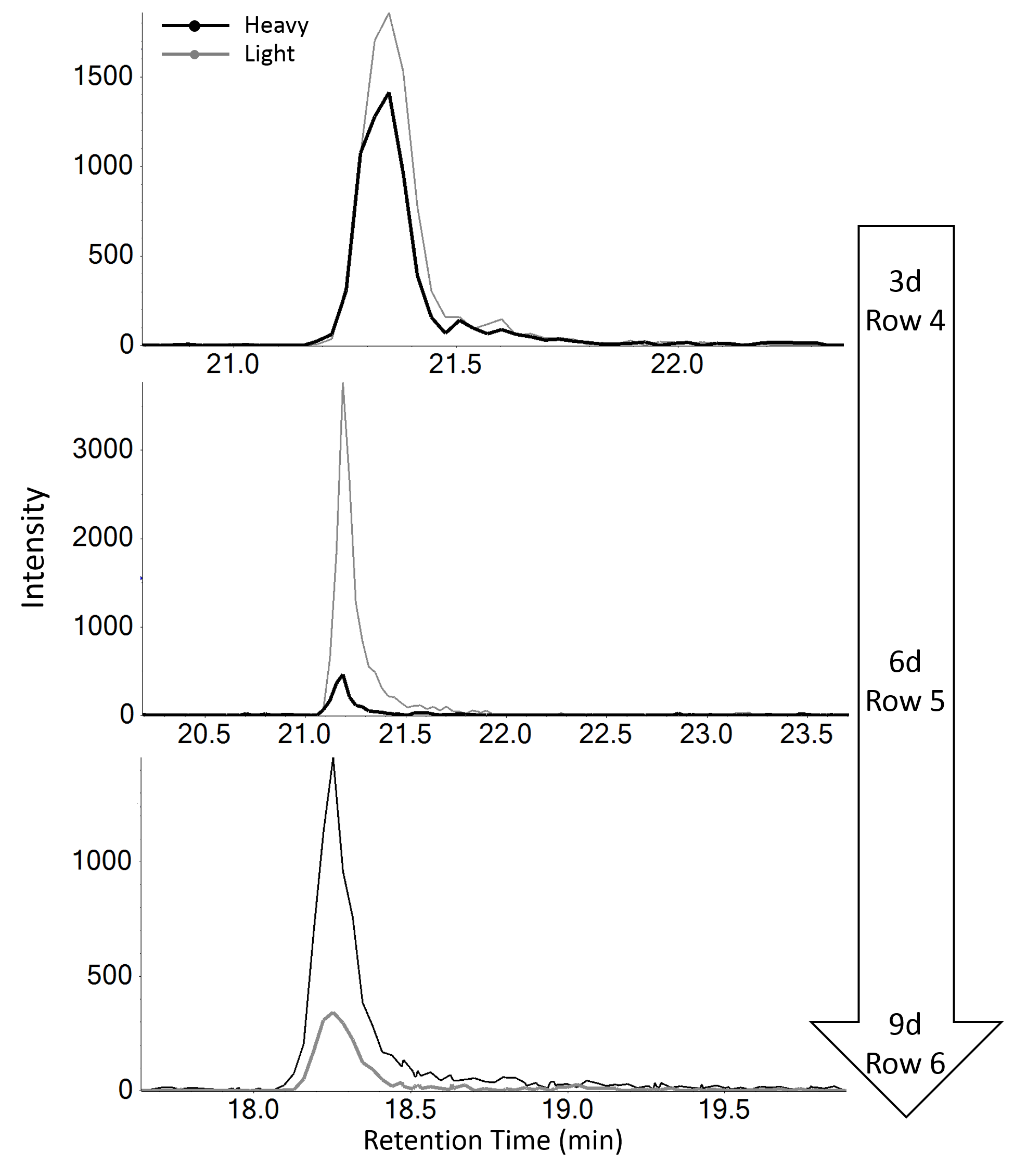


**Figure S5:** The XICs of the reference peptide VGIYFGMK detected in the tryptic digest of the recombinant protein coming from 3-day-old biofilms pulsed with deuterated L-phenylalanine (row 1) or with deuterated L-lysine (row 4), 6-day-old biofilms chased with unlabelled phenylalanine (row 2) or with unlabelled lysine (row 5), and 9-day-old biofilms cross pulsed with deuterated L-lysine (row 3) or with deuterated L-phenylalanine (row 6). Ratios of labelled and unlabelled peptides were calculated based on the peak area of XICs (areas under the curves).


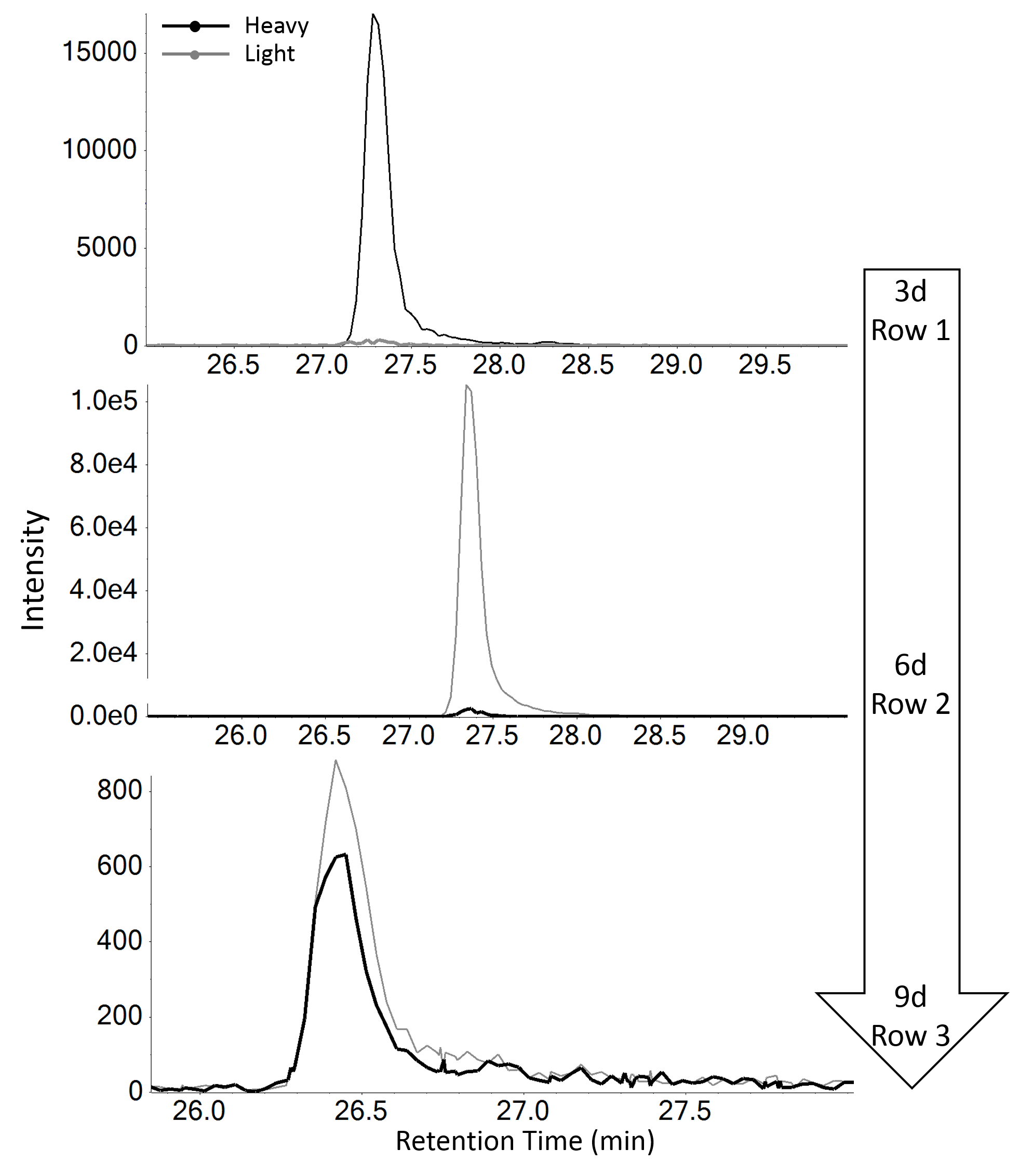

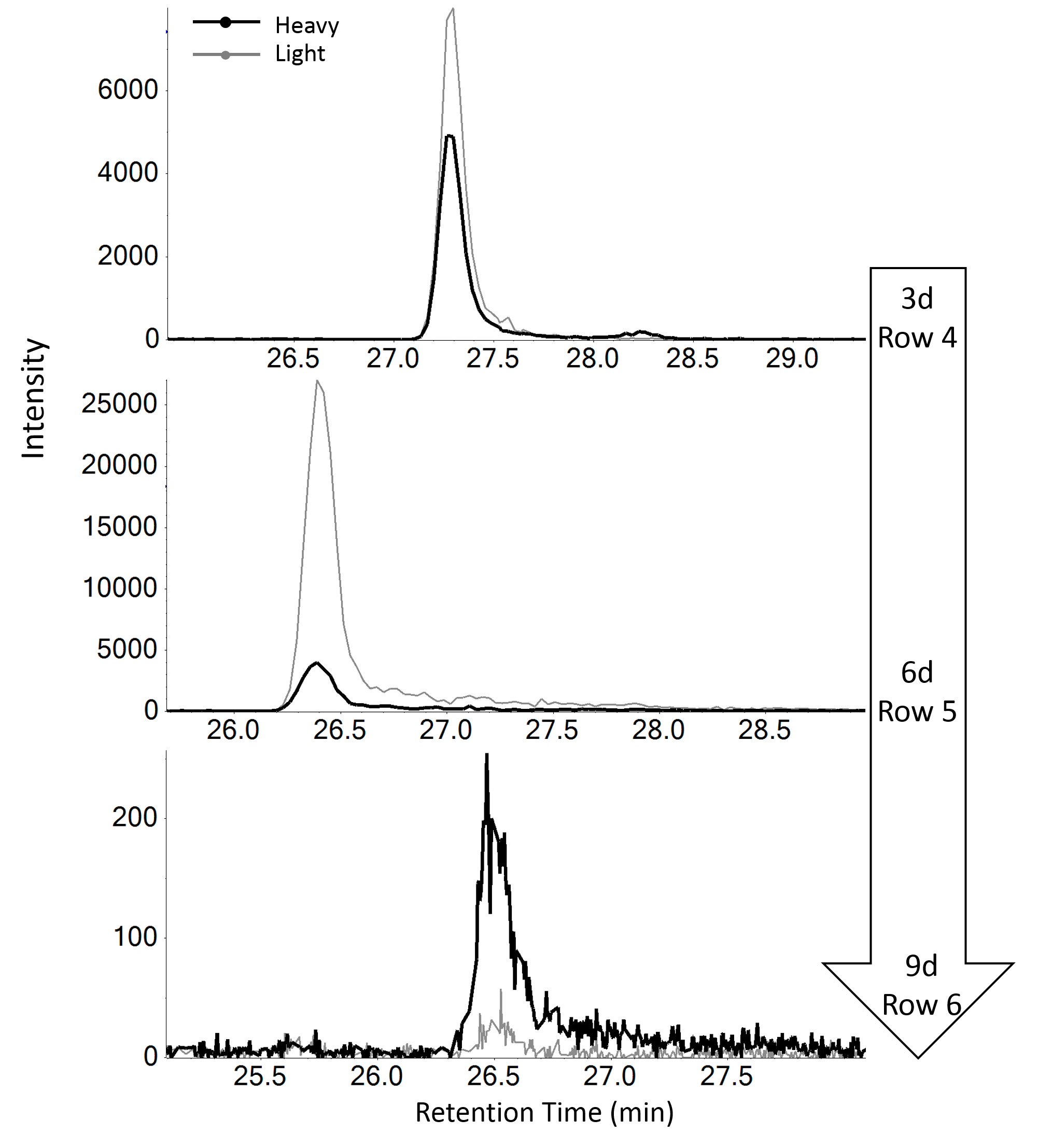


**Figure S6:** The XICs of the peptide DPEFQAQFADLLK detected in the tryptic digest of the recombinant protein coming from 3-day-old biofilms pulsed with deuterated L-phenylalanine (row 1) or with deuterated L-lysine (row 4), 6-day-old biofilms chased with unlabelled phenylalanine (row 2) or with unlabelled lysine (row 5), and 9-day-old biofilms cross pulsed with deuterated L-lysine (row 3) or with deuterated L-phenylalanine (row 6). Ratios of labelled and unlabelled peptides were calculated based on the peak area of XICs (areas under the curves).


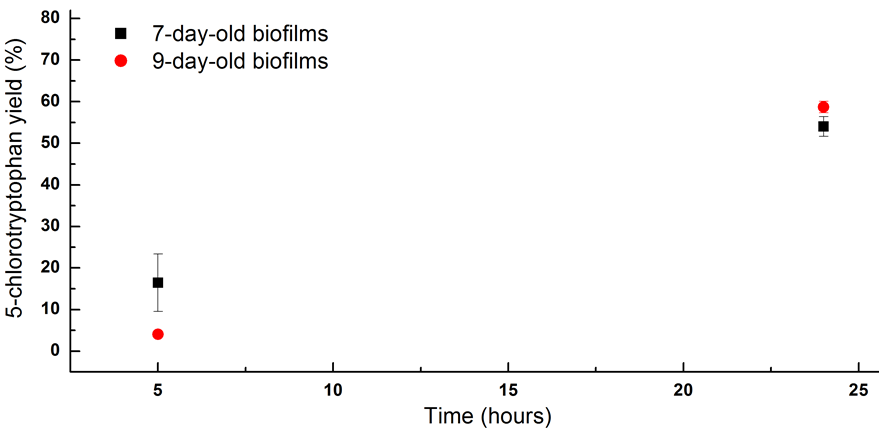


**Figure S7:** Percentage of 5-chlorotryptophan accumulation, monitored by UPLC. Biotransformations of 5-chloroindole were performed by engineered biofilms that had been matured for 7 days (approximately 1.70 mg of total cellular protein present) and 9 days (~1.83 mg of protein), as described in the Methods section in the main manuscript. Concentrations of 5-chlorotryptophan in the biotransformation samples were measured after 5 hours and 24 hours of incubation. Data are mean and s.d. of three experiments.


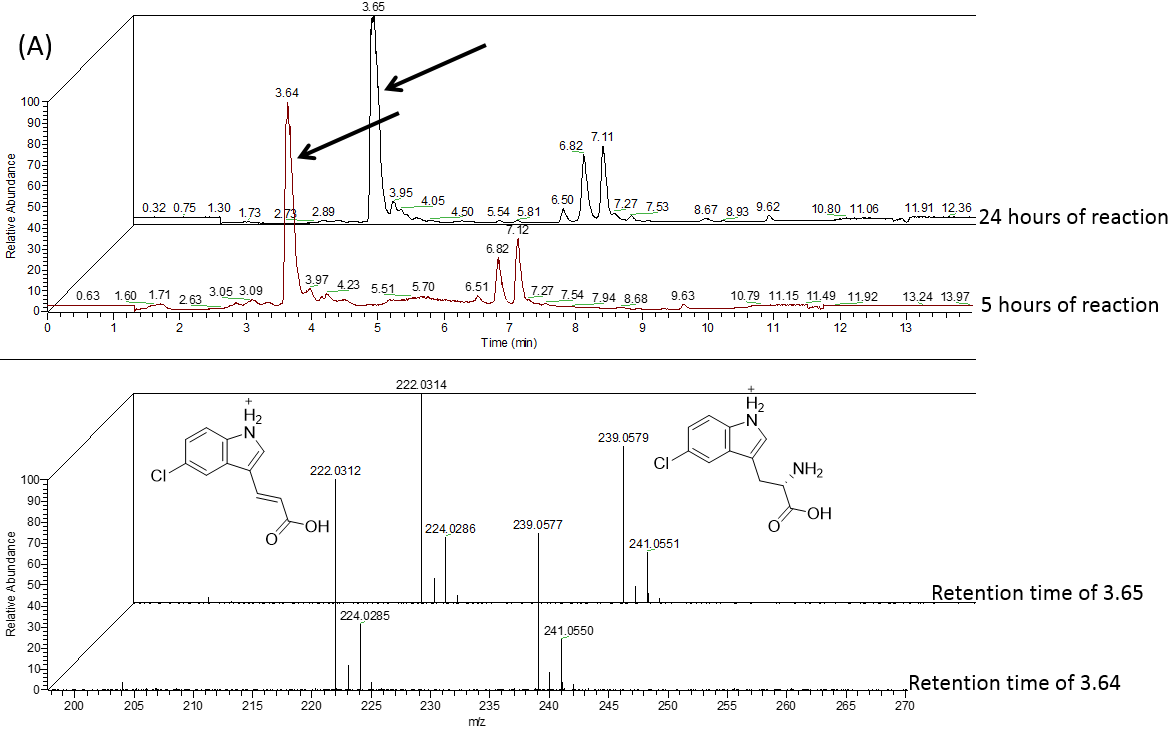


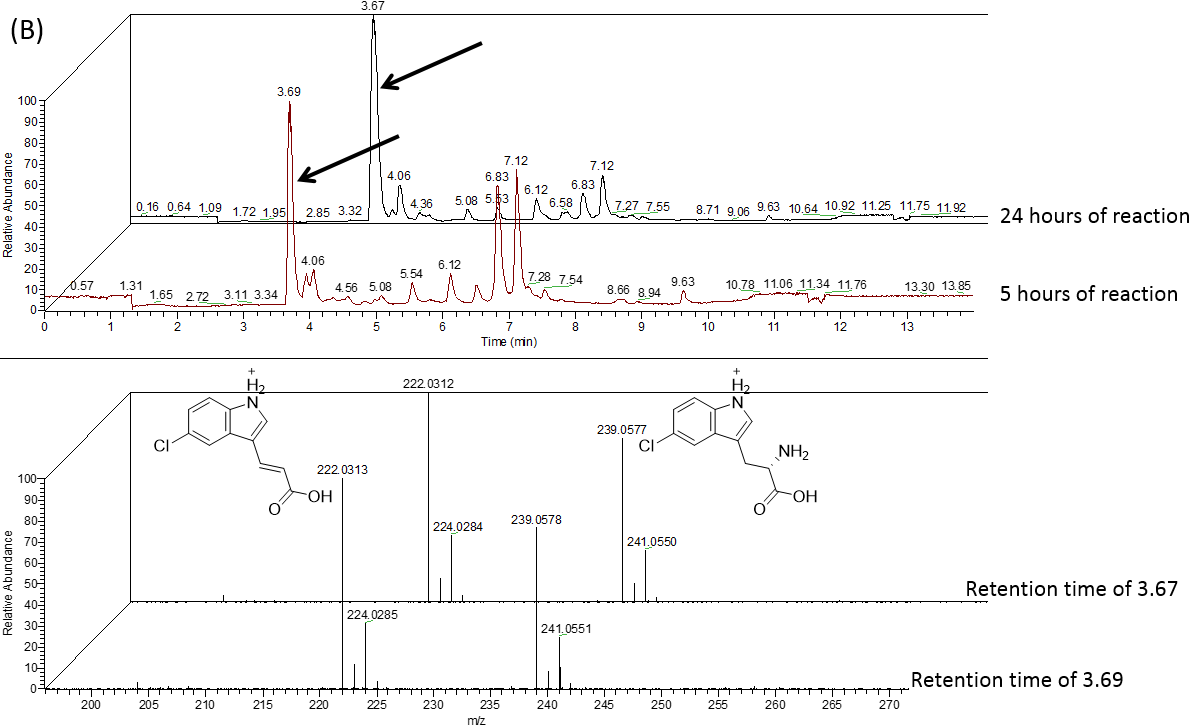


**Figure S8.** Chromatograms and mass spectra of 5-chlorotryptophan generated after incubation of 5-chloroindole with the 7-day-old biofilms (A) and the 9-day-old biofilms (B). Top: MS chromatograms (TICs) of biotransformation samples showing 5-chlorotryptophan (arrowed) (m/z 239.05, RT = ~3.6 mins). Bottom: MS spectra of 5-chlorotryptophan (labelled) (m/z 239.05, eluting at ~3.6 mins). Note: A second m/z 222.03 [M + H]^+^ was the deaminated product of 5-chlorotryptophan, present in both reaction samples and 5-chlorotryptophan standard.

**Reference** 1. Tsoligkas AN, Winn M, Bowen J, Overton TW, Simmons MJ, Goss RJ: **Engineering biofilms for biocatalysis.** *ChemBioChem* 2011, **12:**1391-1395.
